# Supplementary material for: Zinc Laurate Protects against Intestinal Barrier Dysfunction and Inflammation Induced by ETEC in a Mice Model
Source: Nutrients. 2022 Dec 22;15(1):54. doi: 10.3390/nu15010054 (PMC9824434; doi:10.3390/nu15010054)
Supplement: Supplementary file 1 [file nutrients-15-00054-s001.zip › nutrients-2040990-supplementary.pdf]

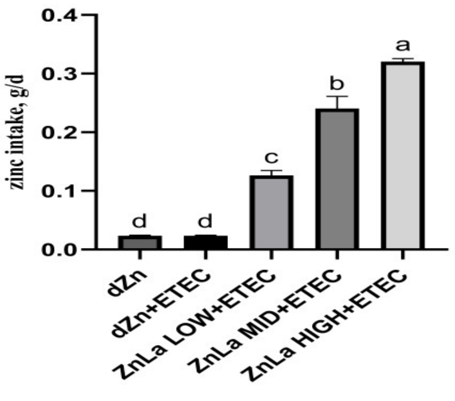

Supplementary Figure S1. Effects of ZnLa on average daily zinc intake in mice. The data were expressed by mean ± SEM (n=9), and different lowercase letters in each group showed significant differences (P < 0.05).

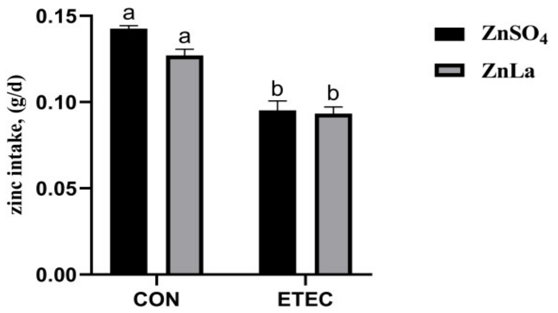

Supplementary Figure S2. Effects of different zinc sources on average daily zinc intake in mice. The data were expressed by mean ± SEM (n=9), and different lowercase letters in each group showed significant differences (P < 0.05).

Supplementary Table S1. Ingredients and composition of marginal zinc deficient diet.

Ingredients	Unit	
Egg White, Spray Dried	%	20.00
Corn starch	%	15.00
Sucrose	%	50.26
Cellulose	%	5.00
Corn oil	%	5.00
Choline bitartrate	%	0.2
Vitamin premix*		
Vitamin A (Acetate)	IU/kg	4000
Vitamin D3 (Cholecalciferol)	IU/kg	1000
Vitamin E (Acetate)	IU/kg	750
Vitamin K3 (Menadione)	mg/kg	0.75
Vitamin B1 (Thiamine hydrochloride)	mg/kg	6.0
Vitamin B2 (Riboflavin)	mg/kg	6.0
Vitamin B3 (Nicotinic acid)	mg/kg	30
Vitamin B5 (D-Calcium pantothenate)	mg/kg	16
Vitamin B6 (Pyridoxine ydrochloride)	mg/kg	7.0
Vitamin B9 (Folic acid)	mg/kg	2.0
Vitamin B12 (Cyanocobalamin)	mg/kg	0.025
D-Biotin	mg/kg	0.2
Minerals premix*		
Ca	g/kg	5
P	g/kg	1.56
K	g/kg	3.6
S	g/kg	0.3
Mg	g/kg	0.5
Cu	mg/kg	6.0
Fe	mg/kg	37
I	mg/kg	0.2
Mn	mg/kg	10.5
Se	mg/kg	0.2
Nutrients content		

Carbohydrate	%	66
Fat	%	5.0
Protein	%	20
Zinc levels	mg/kg	7.0

*Minerals and vitamins were custom designed by Xietong Shengwu Co. LTD (Nanjing, China) according to AIN-93.

Ca is provided as calcium carbonate; P is provided as potassium phosphate monobasic; K is provided as potassium phosphate monobasic, potassium citrate monohydrate and potassium sulfate; S is provided as potassium sulfate; Mg is provided as magnesium oxide; Cu is provided as copper carbonate; Fe is provided as ferric citrate; I is provided as sodium iodide; Mn is provided as manganese carbonate; Se is provided as Sodium selenide.

Supplementary Table S2. Primer set for real-time RT-PCR analysis

Gene	5'–3' Primer sequence
Gapdh	F:TTGAGGTCAATGAAGGGGTC
	R:TCGTCCCGTAGACAAAATGG
Occludin	F:TGGCAAGCGATCATACCCAGAG
	R:CTGCCTGAAGTCATCCACACTC
Claudin-1	F:TGGCTTCTCTGGGATGGATCGG
	R:CCTGAGCGGTCACGATGTTGTC
MUC-2	F:ATCCTCGACGCCTGTGACCTC
	R:GCTGCCCGCTGATGAAGTGAC
ZO-1	F:CCATTCAGGTCGCTCGCATGAC
	R:CATTGCTGGGCTGCTGTGGAG
IL-1β	F:GCAACTGTTCCTGAACTCAACT
	R:ATCTTTTGGGGTCCGTCAACT
IL-6	F:TAGTCCTTCCTACCCCAATTTCC
	R:TTGGTCCTTAGCCACTCCTTC
TNF-α	F:CCCTCACACTCAGATCATCTTCT
	R:GCTACGACGTGGGCTACAG
MyD88	F:ATCGGCTTAAGTTGTGTGTG
	R:AATCGTCAGAAACAACCACC
NF-κB	F:TAACAGCAGGACCCAAGGAC
	R:AGCCCCTAATACACGCCTCT
TLR-4	F:ACCTGGAATGGGAGGACAAT
	R:GTCCAAGTTGCCGTTTCTTG
GPR39	F:ATACCTGCGTAACCCTGATGG
	R:CATGTTCCTGAATTTGAGGTGGC
NHE3	F:TTGGCCGCCTTCTTATTCTGG
	R:TGAAAAGCAGGACAAGGAAATCT

CFTR	F:CTGGACCACACCAATTTTGAGG
	R:GCGTGGATAAGCTGGGGAT

Supplementary Table S3. Primer set for virulence factors analysis

Gene	5'–3' Primer sequence
gapA	F:CGTTAAAGGCGCTAACTTCG
	R:ACGGTGGTCATCAGACCTTC
eltA	F:TTGGTGATCCGGTGGGAAAC
	R:AGGAGGTTTCTGCGTTAGGTG
eltB	F:CACGGAGCTCCCCAGACTAT
	R:GCCTGCCATCGATTCCGTAT
estB	F:TGCCTATGCATCTACACAA
	R:CTCCAGCAGTACCATCTC
luxS	F:CAGTGCCAGTTCTTCGTTGC
	R:TGAACGTCTACCAGTGTGGC
bssS	F:TCCCTTCCTGCTCGGACTTA
	R:CAGACTCATCCGCTCGTAGG
tnaA	F:CGCCAAGAAAGATGCGATGG
	R:CGTCATACAGACCTACCGCC
motA	F:TGAACGACCCCCATTACAGC
	R:AGCGGTCACATGAACACCTT
faeG	F:ACTCAGAAAACCTGATGGTGAAACT
	R:CCCCACCTCTCCCTAACACA
